# Human liver mesenchymal stem/progenitor cells inhibit hepatic stellate cell activation: in vitro and in vivo evaluation

**DOI:** 10.1186/s13287-017-0575-5

**Published:** 2017-06-05

**Authors:** Mustapha Najimi, Silvia Berardis, Hoda El-Kehdy, Valérie Rosseels, Jonathan Evraerts, Catherine Lombard, Adil El Taghdouini, Patrick Henriet, Leo van Grunsven, Etienne Marc Sokal

**Affiliations:** 1 Université Catholique de Louvain, Institut de Recherche Expérimentale et Clinique (IREC), Laboratory of Pediatric Hepatology and Cell Therapy, Avenue Mounier, 52, 1200 Brussels, Belgium; 2 Liver Cell Biology Lab, Vrije Universiteit Brussel (VUB), Laarbeeklaan 103, 1090 Brussels, Belgium; 3 Cell Biology Unit, de Duve Institute, Université Catholique de Louvain, Avenue Hippocrate 75, 1200 Brussels, Belgium

**Keywords:** Liver, Liver fibrosis, Liver stem/progenitor cells, Hepatic stellate cells, Secretome

## Abstract

**Background:**

Progressive liver fibrosis leads to cirrhosis and end-stage liver disease. This disease is a consequence of strong interactions between matrix-producing hepatic stellate cells (HSCs) and resident and infiltrating immune cell populations. Accumulated experimental evidence supports the involvement of adult-derived human liver mesenchymal stem/progenitor cells (ADHLSCs) in liver regeneration. The aim of the present study was to evaluate the influence of ADHLSCs on HSCs, both in vitro and in vivo.

**Methods:**

Activated human HSCs were co-cultured with ADHLSCs or ADHLSC-conditioned culture medium. The characteristics of the activated human HSCs were assessed by microscopy and biochemical assays, whereas proliferation was analyzed using flow cytometry and immunocytochemistry. The secretion profile of activated HSCs was evaluated by ELISA and Luminex. ADHLSCs were transplanted into a juvenile rat model of fibrosis established after co-administration of phenobarbital and CCl_4_.

**Results:**

When co-cultured with ADHLSCs or conditioned medium, the proliferation of HSCs was inhibited, beginning at 24 h and for up to 7 days. The HSCs were blocked in G0/G1 phase, and showed decreased Ki-67 positivity. Pro-collagen I production was reduced, while secretion of HGF, IL-6, MMP1, and MMP2 was enhanced. Neutralization of HGF partially blocked the inhibitory effect of ADHLSCs on the proliferation and secretion profile of HSCs. Repeated intrahepatic transplantation of cryopreserved/thawed ADHLSCs without immunosuppression inhibited the expression of markers of liver fibrosis in 6 out of 11 rats, as compared to their expression in the vehicle-transplanted group.

**Conclusions:**

These data provide evidence for a direct inhibitory effect of ADHLSCs on activated HSCs, which supports their development for the treatment of liver fibrosis.

**Electronic supplementary material:**

The online version of this article (doi:10.1186/s13287-017-0575-5) contains supplementary material, which is available to authorized users.

## Background

Control of liver fibrosis is an unmet medical need. Fibrosis occurs in response to chronic liver injury, and if uncontrolled, leads to cirrhosis and end-stage liver disease [1, 2]. Patients with liver fibrosis require liver transplantation and face long waiting times with progressive disability [3]. Even after successful transplant, advanced fibrosis of the graft can occur [4]. Despite an improved understanding of the mechanisms underlying the development of liver fibrosis, the efficacy of most drugs has not yet been proven in humans [1, 5, 6].

Hepatic stellate cells (HSCs) are the main extracellular matrix-producing cells in the liver. Following injury, HSCs undergo an activation process characterized by the adoption of proliferative, contractile, and fibrogenic myofibroblastic features [7], a process that can be reproduced in plastic culture dishes [8]. The ideal anti-fibrotic therapy should modulate HSC activation, inhibit collagen synthesis, and enhance matrix degradation [9]. Mesenchymal stem cells (MSCs) have been proposed for the treatment of fibrosis based on their hepatocyte differentiation and regeneration potential as well as their immunomodulatory properties [10–13]. Adult-derived human liver stem cells (ADHLSCs) are a subtype of MSCs, which can be obtained by collagenase digestion of the liver, with a preferential hepatocyte differentiation pattern [14, 15]. Like other MSCs, ADHLSCs can produce trophic growth factors and cytokines that are able to overwhelm inflammatory responses [16, 17]. Moreover, MSCs have the potential to exert a suppressive effect on immune cells [18, 19]. ADHLSCs are currently in clinical development, and have been safely and successfully infused in children with inborn errors of liver metabolism [11, 12, 20].

Extrahepatic MSCs were reported to reverse fibrosis in animal models of induced liver fibrosis [21, 22], with frequent improvement of hepatic function. However, the underlying mechanisms are not yet well understood. Therefore, in vitro models and further experimentation are required to better understand how MSCs modulate HSC activation and their paracrine properties [23–25]. Ideally, these systems should use human-derived cells since rodent cells may behave and react differently.

In the current study, we investigated the ability of ADHLSCs to modulate the activation of HSCs both in vitro and in vivo. We demonstrated that ADHLSCs efficiently inhibited HSC proliferation and collagen secretion in vitro, mainly via HGF. Increased secretion of other anti-fibrotic factors by treated HSCs was also noted. In vivo, we showed that repeated intrahepatic transplantation of ADHLSCs was correlated with a decrease in the expression of markers related to liver fibrosis. Thus, our data support the further development of ADHLSCs for the treatment of liver fibrosis.

## Methods

### ADHLSC and HSC isolation and culture

This study was approved by the ethics committee of our institution. Liver tissue was obtained from a Ministry of Health-approved hospital tissue bank (Table 1). ADHLSCs were obtained after primary culture of the parenchymal fraction cells, as previously described [14, 17]. The supernatant, containing the non-parenchymal cell fraction, was processed by Nycodenz gradient (Myegaard, Oslo, Norway) centrifugation to isolate HSCs [26]. Both cell types were cultured in Dulbecco’s modified Eagle’s medium (DMEM) containing 4.5 g/L glucose (Life Technologies, Carlsbad, CA, USA) supplemented with 10% fetal calf serum (FCS; Life Technologies) and 1% penicillin/streptomycin (Life Technologies), at 37 °C in a humidified atmosphere containing 5% CO_2_, as previously described [17]. When the cells reached 80% confluence, they were detached by treatment with 0.05% Trypsin-EDTA (Life Technologies). The viability of the recovered cells was evaluated using the trypan blue exclusion assay.

**Table 1:** Characteristics of the four liver donors whose samples were used for the isolation of HSCs and ADHLSCs used in the current study

Donor number	Age	Gender	Cause of death	Blood group	Ischemia time
89	3 days	M	Respiratory	A+	4 h
93	2 years	F	Metabolic disease (liver transplanted)	O+	1 h 43 min
98	7 days	M	Cardio-respiratory arrest	O-	4 h 20 min
105	46 years	F	Trauma	B+	9 h 43 min

### Co-culture systems

To evaluate the interactions between ADHLSCs and activated HSCs, indirect co-culture in Transwells was used. HSCs were seeded in the lower chamber of a six-well plate at a density of 1.0 × 10^5^ cells/cm^2^, and the ADHLSCs were placed on the collagen-coated membrane inserts (24 mm diameter, 0.4 μm pore size; Corning, Inc., Corning, NY, USA), at ADHLSC:HSC ratios of 1:100, 1:10, 1:1, and 0:1. HSCs were collected at 1, 4, and 7 days for analysis. Cell numbers and viability were evaluated using microscopy and the trypan blue exclusion assay, respectively. Viability was also estimated with a sensitive colorimetric assay, the Cell Counting Kit-8 (CCK-8; Sigma-Aldrich, St. Louis, MO, USA) according to the manufacturer’s instructions.

### Flow cytometry

For cell cycle analysis, 2.0 × 10^5^ cells were used for each experimental condition. Cell suspensions were washed twice with PBS and fixed with 700 μL of cold ethanol for 30 min on ice. The fixed HSCs were washed with PBS, incubated with 100 μg/mL propidium iodide (Life Technologies), 0.1 mg/mL RNase (Sigma-Aldrich), and 0.1% Triton X-100 (Sigma-Aldrich) for 30 min at 37 °C, and then incubated on ice for 15 min. Apoptosis was assessed using the FITC Annexin V Apoptosis Detection Kit I (BD Pharmingen, San Diego, CA, USA) according to the manufacturer’s instructions. Cells treated with 1 mM hydrogen peroxide were used as a positive control. Cells were evaluated with a CANTO II flow cytometer, and the data were analyzed with BD FACSDiva software. The number of cells in different phases of the cell cycle was determined by measuring the area under the curve using FlowJo software (Tree Star, Inc., Ashland, OR, USA).

### Immunocytochemistry

After 24 h of incubation with ADHLSC- or HSC-conditioned medium in 24-well plates, HSCs were fixed with paraformaldehyde (3.5%) for 15 min at room temperature. Immunostaining was performed as previously described [17]. Cells were incubated with a Ki-67 antibody (1:150 dilution; Dako, Glostrup, Denmark) for 1 h. For each experimental condition, four different fields were analyzed, and the number of stained/unstained nuclei (for a total of 2500 nuclei) were counted using ImageJ software (US National Institutes of Health, Bethesda, MD, USA).

### ELISA

After removal of the inserts containing ADHLSCs and aspiration of the medium, recovered HSCs were washed with sterile PBS and incubated in serum-free medium (DMEM containing 4.5 g/L glucose [Life Technologies]) for 24 h. Then, the supernatant was collected, and cells were detached for counting and evaluation of viability. Collagen secretion was evaluated using an ELISA kit for procollagen type I C-Peptide (Takara Bio Inc., Shiga, Japan). The absorbance at 450 nm was measured with a VICTOR *X*4 Plate Reader (Perkin Elmer, Waltham, MA, USA). The results were randomized according to the number of cells collected.

### Western blotting

Total protein lysates were obtained by disrupting 100 mg of rat liver in RIPA buffer [50 mM Tris-Base, pH 8.0, 150 mM NaCl, 1% Triton X-100, 0.5% sodium deoxycholate, and 0.1% SDS, with a protein inhibitor cocktail without EDTA (Roche, Basel, Switzerland)] using FastPrep lysing matrix A beads (MP Biomedicals, Santa Ana, CA, USA) at 6 m/s for 30 s. The protein lysates were incubated on ice for 30 min prior to sample clarification by centrifugation (30 min at maximum speed). Then, sample supernatants were collected, and total protein was quantified using the BCA quantification kit (Thermo Fisher Scientific, Waltham, MA, USA).

Western blotting was performed as previously described [27]. Briefly, extract containing 50 μg of total protein was mixed with loading buffer [Tris-HCl (pH 6.8), glycerol, SDS, DTT, and bromophenol blue], denatured by incubation at 95 °C for 5 min, and then loaded on a 10% Tris-glycine SDS-PAGE gel for protein separation. The separated proteins were transferred onto PVDF membranes overnight at 4 °C. Membranes were incubated with 5% BSA blocking solution for 60 min at room temperature, and then with the primary antibodies (see Table 2) overnight at 4 °C. Then, membranes were thoroughly washed with TBS-T, and incubated with fluorescently labelled secondary antibodies (Table 2) for 60 min at room temperature. The membranes were washed three times in PBS-T, and detected with a Li-cor scanner (Odyssey; Li-cor Biosciences, Lincoln, NE, USA).

**Table 2:** Antibodies used for western blotting analysis

	Reference	Concentration used	Supplier
*Primary*			
Anti-α-SMA	M0851	1/250	Dako
Anti-fibronectin	Ab2413	2 μg/ml	Abcam
Anti-GAPDH	MAB374	1/5000	Millipore
*Secondary*			
Donkey anti-rabbit	BTIU20418	1/10000	Biotium
Donkey anti-mouse	BTIU20363	1/10000	Biotium

### Luminex analysis

After 24 h of co-culture, the secretome of HSCs was evaluated using the 9-plex kit, including tumor necrosis factor alpha (TNFα), metalloproteinase 9 (MMP-9), metalloproteinase 2 (MMP-2), interferon gamma (IFN)-γ, hepatocyte growth factor (HGF), interleukin 6 (IL-6), metalloproteinase 1 (MMP-1), interleukin 10 (IL-10), and metalloproteinase 13 (MMP-13) (R&D Systems, Minneapolis, MN, USA) and Luminex technology (Bio-Plex 200; Bio-Rad Laboratories, Hercules, CA, USA). The supernatants were prepared as previously described for the pro-collagen type I C-Peptide ELISA. The assays were performed according to the manufacturer’s instructions. The data were analyzed using Bio-Plex Manager 6.0 (Bio-Rad Laboratories).

### HGF neutralization

HGF was neutralized with an anti-HGF antibody (20 μg/mL; R&D Systems) diluted in conditioned medium. The conditioned medium without the anti-HGF antibody was used as a control. The antibody was incubated in ADHLSC- or HSC-conditioned medium for 1 h at 37 °C before adding to HSCs. After 24-h incubation, HSCs were harvested for counting, viability assays, and secretome analysis.

### Induction of liver fibrosis in young rats

All animal experiments were performed in compliance with Belgian law for animal protection and were approved by the local ethics review board. All ADHLSCs used in the current study were cryopreserved at the fifth passage. On-site newborn Wistar-Han rats (Charles River, Lyon, France) were intraperitoneally injected with phenobarbital (30 mg/kg^−1^/day^−1^) until day 4. Then, phenobarbital was added to the drinking water (0.5 g/L) until the end of the experiment. On postnatal day 8, CCl_4_ (diluted in corn oil) was subcutaneously injected twice a week at increasing concentrations (0.2–0.5 mL/kg) until 2 days post-cell transplantation.

### Cell transplantation

At 3 weeks old, ADHLSCs (12.5 × 10^6^ per kg; viability >90% by trypan blue exclusion assay) were intrahepatically transplanted into male rats once a week for 2 or 3 weeks under isoflurane (1.5%) anesthesia. The rats were followed for 3 days post-transplantation. The animals were sacrificed on postnatal day 48. Liver samples were recovered for mRNA and protein analyses (n = 6–8 per group).

### Statistics

Results are expressed as mean ± standard error of the mean (SEM). Statistical differences were determined by paired Student’s *t* test for analysis of two samples and one-way ANOVA followed by the Newman-Keuls post hoc test. Differences were considered significant at *p* values less than 0.05 (^*^), 0.01 (^**^), and 0.001 (^***^).

## Results

### ADHLSCs inhibit both the adhesion and proliferation of HSCs

**Fig. 1:**
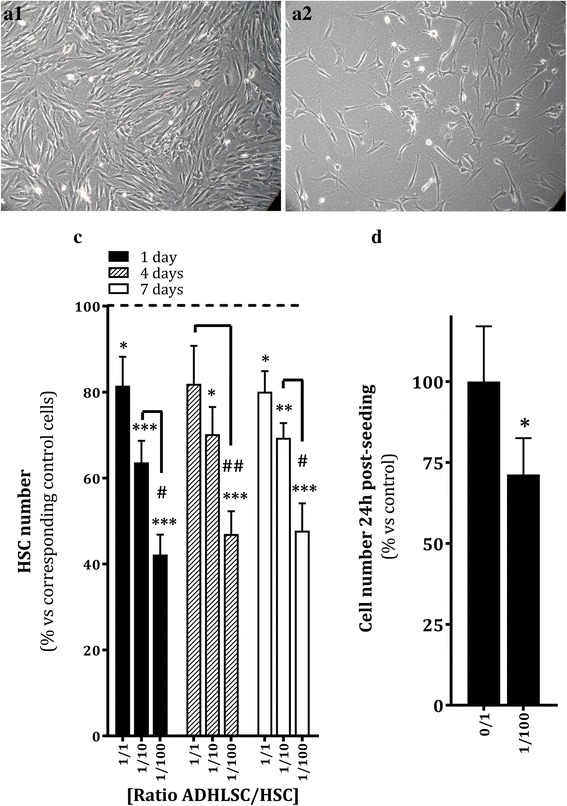
Effect of ADHLSCs on HSC culture. **a** Morphology of HSCs recovered after 24 h of co-culture without ADHLSCs (A1) or with ADHLSCs at an ADHLSC:HSC ratio of 1:100 (A2). **b** Significant decrease in the number of adherent HSCs after 24 h of co-culture at an ADHLSC:HSC ratio of 1:100, which was maintained for up to 7 days (N = 6 for 24 h; N = 4 for 4 and 7 days). **c** Significant decrease in the number of HSCs after 24 h of co-culture at an ADHLSC:HSC ratio of 1:100, using the CCK-8 biochemical assay (N = 4). Results are expressed as mean ± standard error of the mean (SEM). ^***^ *p* value < 0.001; ^**^ *p* < 0.01; ^*^ *p* < 0.05. *ADHLSCs* adult-derived human liver mesenchymal stem/progenitor cells, *HSC* hepatic stellate cells

To investigate the effects of ADHLSCs on activated HSCs, we used the Transwell co-culture system. Activated HSCs are characterized by an increased proliferation rate; therefore, we first analyzed the impact of co-culture on HSC yield. HSCs were plated at a fixed concentration of 1.0 × 10^4^ cells/cm^2^ in the lower chamber, and ADHLSCs were seeded on the collagen-coated membrane insert at different densities to obtain ratios of 0:1, 1:100, 1:10, and 1:1. Activated HSCs (from four different donors) were collected after 24 h, 4 days, and 7 days of co-culture. A significant decrease in the number of recovered HSCs was noted after 24 h. The greatest decrease was observed at the lowest ADHLSC density (a 1:100 ratio; Fig. 1a, b). This inhibitory effect was maintained at 4 and 7 days (Fig. 1b). Comparative analysis of the proliferation index from day 1 to day 7 among all groups (0:1, 1:1, 1:10, and 1:100) revealed that this coefficient was the same between all analyzed groups, which means that the inhibitory effect was initiated during the first 24 h of co-culture.

To confirm the counting data observed at 24 h of co-culture, we used the CCK-8 assay. Using spectrophotometry, we confirmed that HSCs co-cultured with ADHLSCs at a ratio of 1:100 showed a 30% decrease in cell number (Fig. 1c). Then, we further analyzed the mechanisms behind the reduced number of adhering HSCs obtained after 24 h of co-culture at a 1:100 ratio. We investigated whether this could be related to poor adherence, inhibition of proliferation, and/or induced cell death. We first demonstrated that the decreased number of adherent HSCs was correlated with a significant, fourfold greater number of cells in the floating fraction in the culture supernatant (Additional file 1: Figure S1A).

We also checked whether the same effect could be observed with ADHLSC-conditioned medium. Both ADHLSCs and HSCs (as controls) were seeded separately, at the same initial density as was used in the co-cultures. Twenty-four hours later, the culture supernatants were collected and incubated with HSCs for 24 h. The described effect was only observed when conditioned medium from ADHLSCs was used (Additional file 1: Figure S1B). We also observed that the inhibitory effect of ADHLSCs (or conditioned medium) on HSCs was maintained even when allogeneic ADHLSCs were used (data not shown). Next, we evaluated whether the ADHLSCs had an effect on the cell death of HSCs by using Annexin V-PI staining and flow cytometry. We did not observe any effect on cell death. The same observations were made for HSCs incubated with ADHLSCs and with conditioned medium. The analysis showed that the majority of the cells were viable (Additional file 1: Figure S1C). To understand the kinetics of this deficient adhesion, we analyzed the number of floating and adhering HSCs at different time points post-seeding in the presence of either ADHLSC- or HSC-conditioned medium. Our results demonstrated that early after seeding, in the presence of ADHLSC-conditioned medium the number of HSCs in the floating fraction is twice as high as the number in the presence of control HSC-conditioned medium (Additional file 2: Figure S2A). This delayed adhesion remained, and it reached a maximum 24 h post-seeding. Likewise, the number of adhering cells was lower when incubated with ADHLSC-conditioned medium (Additional file 2: Figure S2B).

**Fig. 2:**
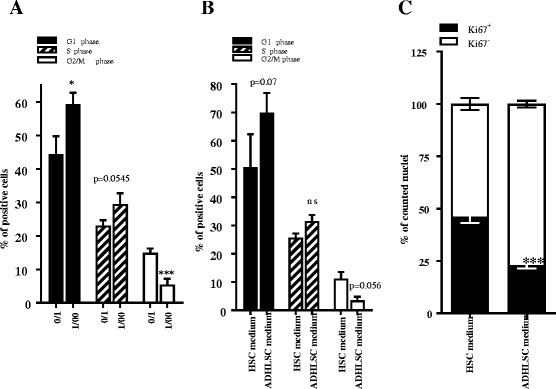
Effect of ADHLSCs on HSC proliferation. Cell cycle analysis of PI-stained HSCs demonstrated an increase in the number of HSC blocked in G0/G1 phase and a decrease in the number of HSCs in G2/M phase after 24 h of co-culture at an ADHLSC:HSC ratio of 1:100 (**a**) and after 24 h of incubation with ADHLSC-conditioned medium (**b**) (N = 4). **c** The Ki67 immunostaining of HSCs showed a significant decrease in the number of immunostained nuclei of HSCs pre-incubated for 24 h with ADHLSC conditioned medium (N = 4). For each experimental condition, four different fields were analyzed, and a total of 2500 nuclei were counted using Image J software. Results are expressed as mean ± standard error of the mean (SEM). ^***^ *p* value < 0.001; ^**^ *p* < 0.01; ^*^ *p* < 0.05. *ADHLSCs* adult-derived human liver mesenchymal stem/progenitor cells, *HSC* hepatic stellate cells

Furthermore, we evaluated the effect of ADHLSCs on the cell cycle of HSCs by PI staining and flow cytometry. We demonstrated that a significantly higher number of HSCs co-cultured for 24 h with ADHLSCs at ratio of 1:100 were blocked in G0/G1 phase when compared to the number in control cultures without ADHLSCs (Fig. 2a). Concomitantly, we observed a significant decrease in the number of HSCs in G2/M phase following co-culture with ADHLSCs (Fig. 2a). The same result was obtained when HSCs were incubated with ADHLSC-conditioned medium (Fig. 2b). By using immunocytochemistry for Ki67, we confirmed a significant decrease in the number of immune-positive HSC nuclei (in four different microscopy fields per donor) after a 24-h incubation with ADHLSC-conditioned medium (Fig. 2c).

### ADHLSCs downregulate HSC secretion of pro-collagen type I and stimulate the release of anti-fibrotic molecules

**Fig. 3:**
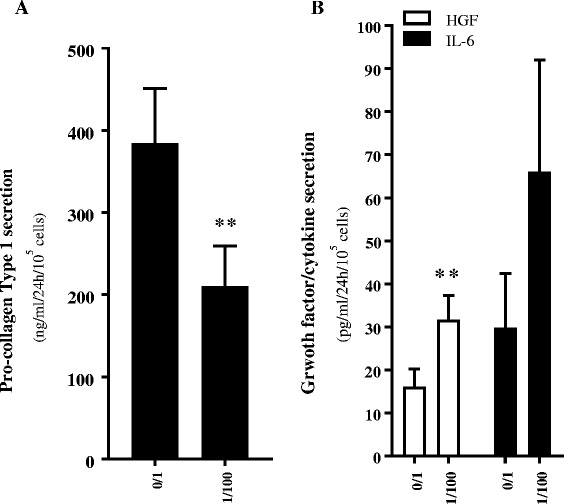
Modulation of the HSC secretome after 24 h of co-culture with ADHLSCs. **a** ADHLSCs inhibit pro-collagen type I secretion by HSCs after 24 h of co-culture at a ADHLSC: HSC ratio of 1:100, as evaluated by ELISA (N = 4). **b** The presence of ADHLSCs increased HGF and IL-6 secretion by HSCs after 24 h of co-culture at an ADHLSC:HSC ratio of 1:100, as evaluated by multiplex technology (N = 4). Results are expressed as mean ± standard error of the mean (SEM). ^***^ *p* value < 0.001; ^**^ *p* < 0.01; ^*^ *p* < 005. *HGF* hepatocyte growth factor, *IL-6* interleukin 6

Upon activation, the secretion of collagen type I by HSCs is increased. Therefore, we investigated the effect of ADHLSCs on the collagen secretion capacity of HSCs by measuring pro-collagen I (a precursor of collagen type I) secretion by ELISA. After 24 h of co-culture, we observed a significant (45%) decrease in the amount of pro-collagen I secreted by HSCs when co-cultured with ADHLSCs (Fig. 3a). Using a multiplex Luminex assay, we also demonstrated that the level of HGF and IL-6 secreted by HSCs was increased when co-cultured with ADHLSCs for 24 h (Fig. 3b). The secreted levels of the metalloproteinases MMP1 and MMP2 were also increased after 24 h of co-culture with ADHLSCs (Table 3).

**Table 3:** Levels of various metalloproteinases secreted by HSCs when co-cultured with or without ADHLSCs for 24 h

	MMP1 (pg/mL^−1^/24 h^−1^/10^5^ cells^−1^)				MMP2 (pg/mL^−1^/24 h^−1^/10^5^ cells^−1^)			
	Donor 1	Donor 2	Donor 3	Donor 4	Donor 1	Donor 2	Donor 3	Donor 4
*ADHLSC to HSC ratio*								
0:1	474.18	1183.05	1005.03	4550.99	329.02	469.68	1919.75	1959.93
1:100	843.57	1515.79	2248.78	6462.41	466.6	718.12	3288.16	2496.38

### Neutralization of HGF reverses the inhibitory effect of ADHLSCs on the number of HSCs and the secretion profile

**Fig. 4:**
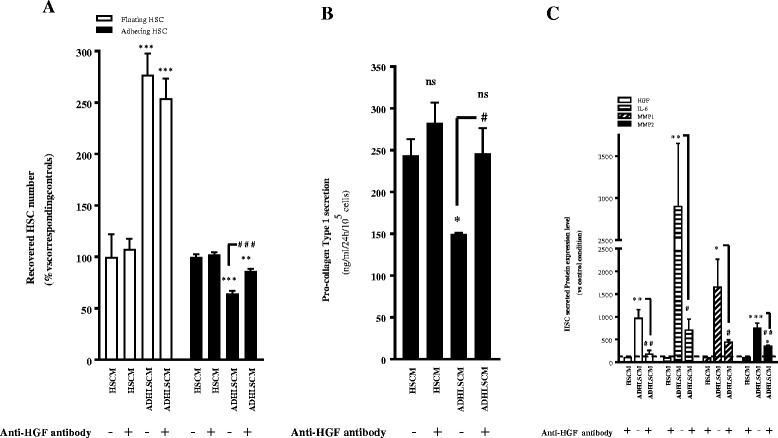
Involvement of HGF in the effect of ADHLSCs on HSC proliferation and procollagen type I secretion. **a** The numbers of floating and adhering HSCs after 24 h of incubation with ADHLSC- or HSC-conditioned medium was evaluated after pre-incubation with or without an anti-HGF antibody. There was significant, partial inhibition of the decrease in the number of adhering HSCs when incubated with ADHLSC-conditioned medium containing anti-HGF antibody (N > 5). **b** Inhibition of the effect of ADHLSC-conditioned medium on HSC pro-collagen type I secretion in the presence of an anti-HGF antibody (N = 3). **c** Anti-HGF antibody significantly inhibited the increase in HGF, IL-6, MMP1, and MMP2 secretion by HSCs. *HGF* hepatocyte growth factor, *HSC* hepatic stellate cells

ADHLSCs secrete high levels of HGF, a growth factor known to have anti-fibrotic properties [17]. As the inhibitory effect of ADHLSCs on HSC proliferation seems to be mediated by soluble factors, we investigated the potential role of HGF by neutralizing this growth factor. HSCs were incubated with conditioned medium that was pre-incubated with an anti-HGF antibody. After 24 h, we did not observe any effect on the floating HSC population (Fig. 4a). Nevertheless, HGF appears to be more involved in modulating the fraction of adherent HSCs. Indeed, when the anti-HGF antibody was diluted in the ADHLSC-conditioned medium, it only partially inhibited the decrease in HSCs (Fig. 4a). We also demonstrated that the anti-HGF antibody blocked the inhibitory effect of ADHLSC-conditioned medium on the secretion of collagen, IL-6, HGF, MMP1, and MMP2 by HSCs (Fig. 4b, c).

### Transplantation of ADHLSCs into a juvenile rat model of liver fibrosis decreases the expression of genes related to HSC activation

**Fig. 5:**
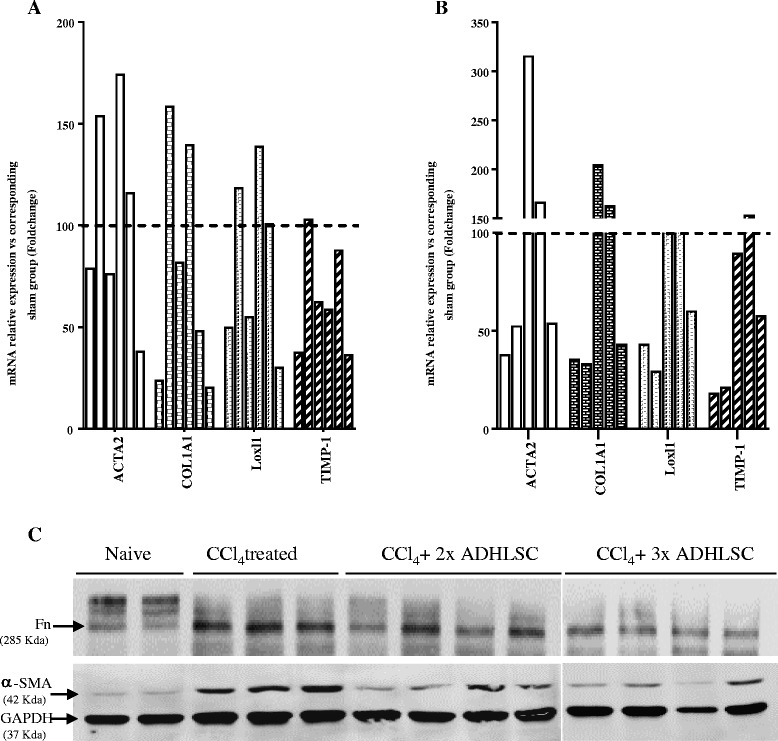
Effect of ADHLSC transplantation on the expression of HSC activation markers in an established model of liver fibrosis. The mRNA levels of αSMA, Col1α1, Lox, and TIMP-1 in the livers of rats intrahepatically transplanted twice (n = 6) (**a**) or three times (n = 5) (**b**) with ADHLSCs without immunosuppression were estimated using RT-qPCR. **c** Total protein extracts were prepared from the three groups of rats as well as from naïve controls (n = 2), and immunoblotted with antibodies against markers of HSC activation and GAPDH as described in the “Materials and Methods” section. Downregulation of α-SMA, and fibronectin (*Fn*) is correlated to ADHLSC transplantation twice (n = 4) and three times (n = 4). *ADHLSCs* adult-derived human liver mesenchymal stem/progenitor cells

To assess whether ADHLSCs could modulate HSC activation in vivo, we transplanted MSCs transhepatically into young rats that were chronically treated with phenobarbital and CCl_4_ [28]. In the first group, ADHLSCs were transplanted twice (once per week) to reach a total number of 25 × 10^6^ transplanted cells/kg body weight, and analyses were performed 3 weeks later (the CCl_4_ treatment was maintained during this time period). In the second group, ADHLSCs were transplanted three times to reach a total number of 37.5 × 10^6^ cells/kg body weight, and the analyses were performed 2 weeks later. The expression of genes related to HSC activation/liver fibrosis was estimated by using RT-qPCR. Our data showed that the expression of α-SMA, collagen 1α1, Loxl1, and TIMP-1 was decreased in three out of six rats in the group that was transplanted twice compared to the levels in vehicle-transplanted rats (Fig. 5a). In rats that were transplanted three times, the expression of such markers was decreased in three out of five rats (Fig. 5b). We also checked the protein expression of HSC activation markers, and observed that α-SMA protein expression was significantly decreased in CCl_4_-treated, cell-transplanted rats (in both groups) as compared to the expression in the CCl_4_-treated, nontransplanted group (Fig. 5c). The same observation was confirmed for another myofibroblastic marker fibronectin (Fig. 5c).We also showed that the rats displaying a decreased α-SMA expression level after ADHLSC transplantation (either two or three times) also exhibited lower concentrations of liver transaminases (Additional file 3).

## Discussion

In the current study, we demonstrated the inhibitory effect of ADHLSCs on HSC activation both in vitro and in vivo. In vitro, we showed that ADHLSCs inhibit the proliferation of HSCs, decrease their pro-collagen production, and induce the secretion of anti-fibrotic molecules. Such effects are mediated via the HGF pathway. Our findings also showed, in an established rat model of liver fibrosis, a decrease in the expression of markers related to liver fibrosis following ADHLSC transplantation.

Because HSCs are the principal mediators of liver fibrosis, several therapeutic approaches have directly targeted their activation. However, the efficacy of the currently developed anti-fibrotic drugs has not been proven in humans [1, 6]. MSC-based therapy for treating liver fibrosis based on the differentiation potential and immunoregulatory properties of MSCs is currently under evaluation [9, 29–31]. Cell-free mechanisms have also been proposed [31–33]. Our study investigated ADHLSC-HSC interactions with an in vitro co-culture system. Indeed, HSC activation is observed in vitro, as evidenced by the progressive loss of vitamin A stores, the increased proliferation rate, and extracellular matrix production when cultured on plastic [2]. In the co-culture system we used, we demonstrated that ADHLSCs could inhibit HSC proliferation after 24 h of co-culture through soluble factors constitutively secreted by ADHLSCs. The most potent effect was observed at an ADHLSC/HSC ratio of 1:100 and was reproduced with ADHLSC-conditioned medium (obtained 24 h after plating, at the same density used in the co-culture) and allogeneic ADHLSCs. The finding that the inhibition of HSC proliferation was inversely proportional to the number of ADHLSCs used is a striking difference compared to observations made with other extrahepatic MSCs [24, 25]. This difference could be explained in one of two ways; first, an inhibitory molecule secreted by ADHLSCs or the HSCs themselves when mixed at higher ADHLSC:HSC ratios, and second, more efficient action of the soluble anti-fibrotic factor(s) at a certain concentration. To further characterize the mechanism of HSC proliferation inhibition, we blocked the secretion of HGF, one of the factors that are highly secreted by ADHLSCs [17]. HGF neutralization partially reversed the inhibitory effect of ADHLSCs on HSC proliferation. These data are in accordance with other published data describing the ability of HGF to inhibit the platelet-derived growth factor (PDGF)-mediated overproliferation of myofibroblasts [34].

The decrease in the amount of pro-collagen type 1 secreted by HSCs is promising, as the collagen type 1 is a major component of the extracellular matrix that is produced in excess during the development of liver fibrosis. HGF neutralization also reversed the ADHLSC-induced inhibition of HSC pro-collagen I secretion, in accordance with previous data showing the ability of HGF to inhibit the profibrogenic transforming growth factor beta (TGF-β) pathway [34]. HGF can induce the production of metalloproteinases by lung myofibroblasts [34], and we showed that HSCs also increased the secretion of MMP1 and 2, which are both involved in extracellular matrix degradation. The increase in the secretion of IL-6 by HSCs in our model could also be of therapeutic importance, as this cytokine has been implicated in the regeneration and survival of hepatocytes [35]. A recent study demonstrated that IL-6 and murine MSCs synergistically enhanced hepatic repair, improved hepatic function, and reduced liver fibrosis in mice [36].

Our in vivo findings showed that after ADHLSC transplantation, mRNA expression of genes related to fibrosis, including α-SMA, Col1α1, Loxl1, and TIMP-1 was decreased in three out of six rats that were infused twice (one infusion per week). This effect was observed 3 weeks after the last cell infusion. In the group of rats transplanted three times, the decreased mRNA expression of the above-mentioned genes was observed in three out of five animals 2 weeks after the last cell injection. Myofibroblast protein markers, including α-SMA and fibronectin which were reported to be upregulated after CCl_4_-induced liver fibrosis in rats [37, 38], were also significantly decreased after ADHLSC transplantation at least in half of the analyzed rats in each group. Although the data from the thrice-transplanted group seemed better, we cannot exclude the fact that the post-transplantation timing of the analysis may explain these potential differences. The group with two injections was analyzed 3 weeks after the last injection, whereas the group with three injections was analyzed only 2 weeks after the last injection.

Both groups were transplanted without immunosuppression, and no human cells were detected in the transplanted rat livers after targeting human Ku-80 via immunohistochemistry (data not shown). These data were in accordance with a recent study claiming that MSCs are cleared in CCl_4_-damaged livers by 7 days post-transplantation [39] and support the cell-free mechanism of transplanted ADHLSCs in that liver fibrosis model.

## Conclusions

The present study investigates the ability of ADHLSCs to modulate the activation of HSCs, a key process involved in liver fibrosis establishment. In summary, obtained data demonstrate that ADHLSCs inhibit HSC proliferation and pro-collagen secretion in vitro via paracrine mechanisms. Increased secretion of other anti-fibrotic factors by HSCs, like IL-6, was also noted upon co-culture with ADHLSC. Those effects seem to be mediated at least in part, by the anti-fibrotic molecule HGF, highly secreted by ADHLSC. The study also shows that repeated intrahepatic transplantation of ADHLSCs in a rat model of liver fibrosis is correlated with a decrease in the expression of HSC activation markers. Taken together, our data support the further development of ADHLSCs and/or their secretome for the treatment of liver fibrosis.

## Additional files

Additional file 1: Figure S1. Effect of ADHLSC on HSC plating efficiency. (A) The counting of floating and adhering HSC number after 24 hours of co-culture with ADHLSC showed an increase in the number of floating HSC concomitant with a decrease in the number of adhering HSC at an ADHLSC/HSC ratio of 1/100 (n = 4). (B) The counting of the number of floating and adhering HSC after 24 hours of incubation with ADHLSC conditioned medium showed similarly an increase in the % of floating HSC and a decrease in the % of adhering HSC, as compared with HSC incubated the same period of time with HSC conditioned medium (n = 4). C, Following annexin V–PI staining, no significant difference in cell death induction was noticed between HSC cultivated for 24 hours with ADHLSC or with HSC conditioned medium (n = 4). Results are expressed as mean ± standard error of the mean (SEM). ^***^Denotes a *p* value <0.001; ^**^ *p* < 0.01; ^*^ *p* < 0.05. (PPT 271 kb)

Additional file 2: Figure S2. ADHLSC delay HSC post-seeding adhesion. The plating kinetic analysis revealed a higher number of floating HSC (A) and a lower number of adhering HSC (B) at 2, 4, 8 and 24 hours post-seeding in the group of HSC incubated with ADHLSC conditioned medium in comparison with the group incubated with HSC conditioned medium (n = 4). Results are expressed as mean ± standard error of the mean (SEM). ^***^Denotes a *p* value <0.001; ^**^ *p* < 0.01; ^*^ *p* < 0.05. (PPT 205 kb)

Additional file 3: Collection of blood samples and measurement of liver enzymes. (DOCX 18 kb)

## Abbreviations

ADHLSCs: Adult-derived human liver mesenchymal stem/progenitor cells
DMEM: Dulbecco’s modified Eagle’s medium
HGF: Hepatocyte growth factor
HSC: Hepatic stellate cells
IL-6: Interleukin 6
MMP1: Metalloproteinase 1, MMP2, Metalloproteinase 2
MSC: Mesenchymal stem cells
