# Supplementary figures and images for: Human liver mesenchymal stem/progenitor cells inhibit hepatic stellate cell activation: in vitro and in vivo evaluation
Source: Stem Cell Res Ther. 2017 Jun 5;8:131. doi: 10.1186/s13287-017-0575-5 (PMC5460523; doi:10.1186/s13287-017-0575-5)

## Slide 1
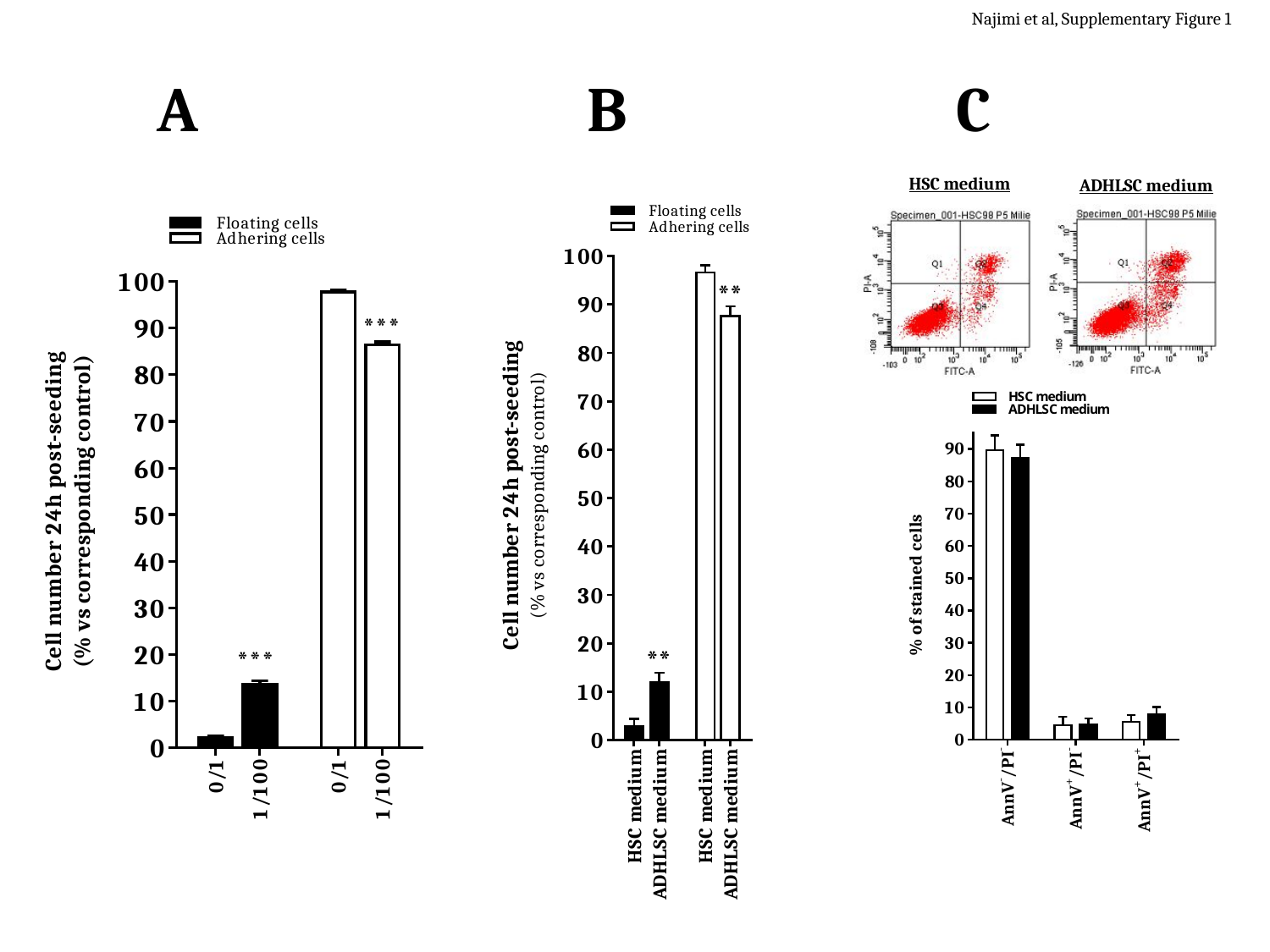

Najimi et al, Supplementary Figure 1
A
B
C
HSC medium
ADHLSC medium

Supplement: Supplementary file 1 — Effect of ADHLSC on HSC plating efficiency. (A) The counting of floating and adhering HSC number after 24 hours of co-culture with ADHLSC showed an increase in the number of floating HSC concomitant with a decrease in the number of adhering HSC at an ADHLSC/HSC ratio of 1/100 (n = 4). (B) The counting of the number of floating and adhering HSC after 24 hours of incubation with ADHLSC conditioned medium showed similarly an increase in the % of floating HSC and a decrease in the % of adhering HSC, as compared with HSC incubated the same period of time with HSC conditioned medium (n = 4). C, Following annexin V–PI staining, no significant difference in cell death induction was noticed between HSC cultivated for 24 hours with ADHLSC or with HSC conditioned medium (n = 4). Results are expressed as mean ± standard error of the mean (SEM). ***Denotes a p value <0.001; ** p < 0.01; * p < 0.05. (PPT 271 kb) [file 13287_2017_575_MOESM1_ESM.ppt]

## Slide 1
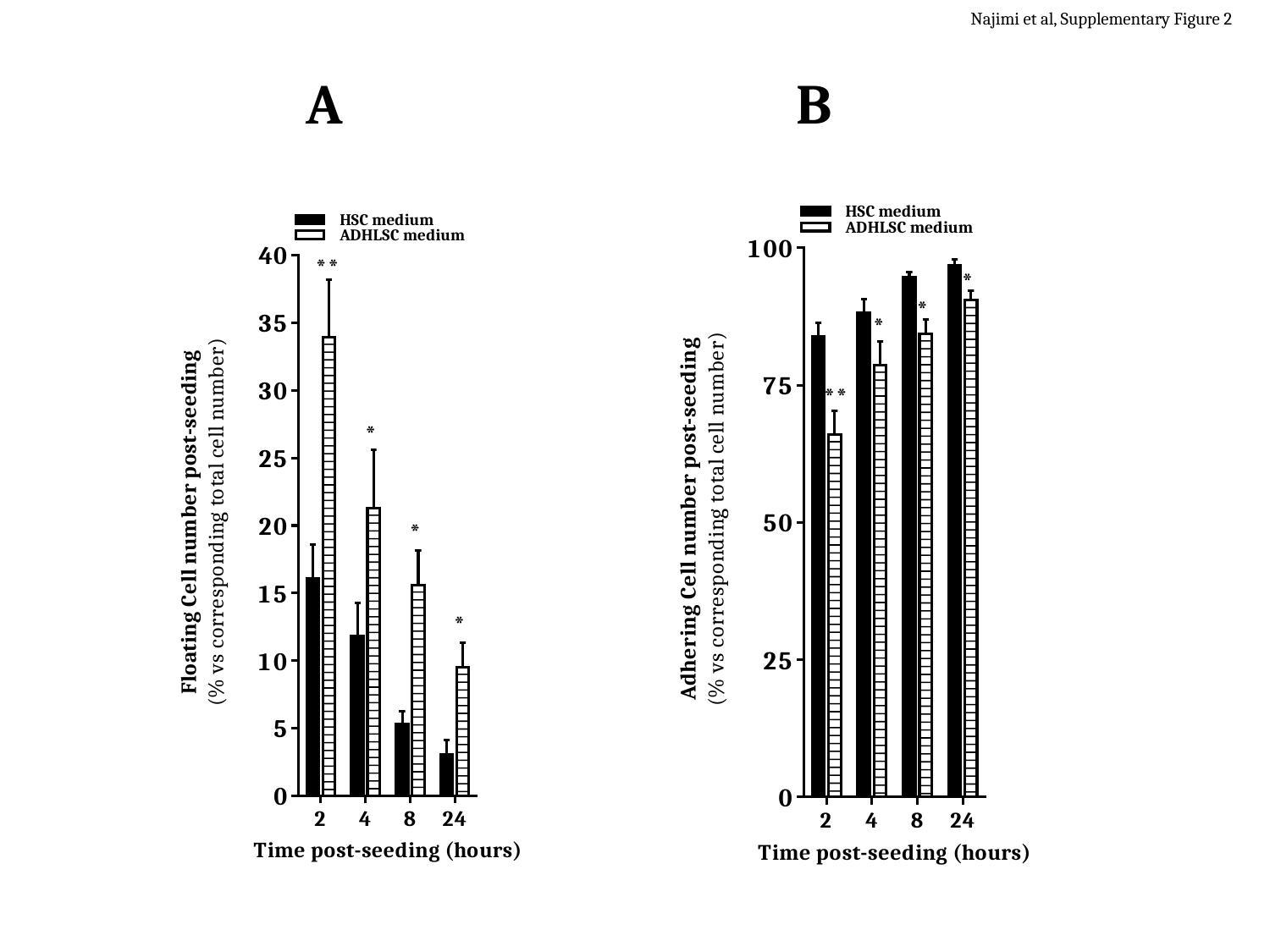

Najimi et al, Supplementary Figure 2
A
B

Supplement: Supplementary file 2 — ADHLSC delay HSC post-seeding adhesion. The plating kinetic analysis revealed a higher number of floating HSC (A) and a lower number of adhering HSC (B) at 2, 4, 8 and 24 hours post-seeding in the group of HSC incubated with ADHLSC conditioned medium in comparison with the group incubated with HSC conditioned medium (n = 4). Results are expressed as mean ± standard error of the mean (SEM). ***Denotes a p value <0.001; ** p < 0.01; * p < 0.05. (PPT 205 kb) [file 13287_2017_575_MOESM2_ESM.ppt]
