# Supplementary material for: Human liver mesenchymal stem/progenitor cells inhibit hepatic stellate cell activation: in vitro and in vivo evaluation
Source: Stem Cell Res Ther. 2017 Jun 5;8:131. doi: 10.1186/s13287-017-0575-5 (PMC5460523; doi:10.1186/s13287-017-0575-5)
Supplement: Supplementary file 3 — Collection of blood samples and measurement of liver enzymes. (DOCX 18 kb) [file 13287_2017_575_MOESM3_ESM.docx]

**Supplementary material**

**Collection of blood samples and measurement of liver enzymes**

Venous blood samples were obtained from the vena cava under 2% isofluorane anesthesia (Zoetis, Belgium), collected in the test tubes and allowed to stand for 30 min to clot before being centrifuged at 10.000 rpm for 10 min twice. The serum was recovered and stored at -80°C for subsequent analysis of alanine aminotransferase (ALT) and aspartate aminotransferase (AST) enzyme levels. The AST and ALT levels were determined according to the method of Reitman and Frankel (1957), using AST and ALT substrate, respectively. Enzyme activity was expressed in International Units per liter (IU/L).

**Supplementary table 1:** Transaminases levels in CCl4 treated rats and subsequently transplanted 2 and times with ADHLSC

|  | **AST**  (IU/L) | **ALT**  (IU/L) |
| --- | --- | --- |
| **Mean Normal value**  **(Charles River Laboratories data)** | 105 | 20 |
| **Mean After CCl4 value (N=8)** | 623 | 490 |
| **CCl4+ ADHLSC 2x individual values** | *351**  752  *328**  609  *323**  *327** | *195**  721  *224**  747  *135**  *209** |
| **CCl4 + ADHLSC 3x individual values** | *384**  *407**  1000  893  *492** | *213**  *215**  1000  854  *162** |

*: rats that have shown a decrease in α-SMA expression

**References:**

Reitman S, Frankel S. A colorimetric method for the determination of serum glutamic oxalacetic and glutamic pyruvic transaminases. Am J Clin Pathol. 1957; 28(1):56-63.
